# Supplementary material for: Lower CSF Amyloid-Beta1–42 Predicts a Higher Mortality Rate in Frontotemporal Dementia
Source: Diagnostics (Basel). 2019 Oct 25;9(4):162. doi: 10.3390/diagnostics9040162 (PMC6963225; doi:10.3390/diagnostics9040162)
Supplement: Supplementary file 1 [file diagnostics-09-00162-s001.docx]

**Supplementary table 1: Comparison of the two groups in terms of vascular risk factors.**

| **Variable** | **Patients Who Died during Follow-Up (*n* = 32)** | **Patients Who Did not Die during Follow-Up (*n* = 67)** | ***p*** |
| --- | --- | --- | --- |
| Atrial Fibrillation, *n* (%) | 1 (3.1) | 3 (4.5) | 0.749 |
| High blood pressure | 9 (28.1) | 35 (52.2) | 0.024 |
| Diabetes | 2 (6.3) | 16 (23.9) | 0.033 |
| Dyslipidemia | 6 (18.8) | 33 (49.3) | 0.004 |
| Obesity | 1 (3.1) | 8 (11.9) | 0.154 |
| Heart failure | 1 (3.1) | 0 (0.0) | 0.146 |
| Coronaropathy | 0 (0.0) | 2 (3.0) | 0.323 |
| Heart valve disease | 1 (3.1) | 3 (4.5) | 0.749 |
| Alcohol abuse | 1 (3.1) | 5 (7.5) | 0.398 |
| Smoking | 0 (0.0) | 1 (1.5) | 0.484 |
| Obstructive sleep apnoea | 0 (0.0) | 3 (4.5) | 0.224 |
| History of stroke | 2 (6.3) | 4 (6.0) | 0.956 |
